# An Update on Uterine Smooth Muscle Tumors: Is It a Leiomyoma, a STUMP, or a Leiomyosarcoma?

**DOI:** 10.3390/biomedicines14020285

**Published:** 2026-01-27

**Authors:** Catalin-Bogdan Satala, Gabriela Patrichi, Alina Mihaela Gurau, Alexandra Toma, Constantin Popazu, Daniela Mihalache

**Affiliations:** 1 Faculty of Medicine and Pharmacy, Medical and Pharmaceutical Research Center, “Dunărea de Jos” University of Galați, 800008 Galati, Romania or stlcatalin92@yahoo.com (C.-B.S.); alexandra.toma@ugal.ro (A.T.); constantin.popazu@ugal.ro (C.P.); daniela.mihalache@ugal.ro (D.M.); 2 Department of Pathology, Clinical County Emergency Hospital, 810325 Brăila, Romania; 3 The Doctoral School of Medicine and Pharmacy, “George Emil Palade” University of Medicine, Pharmacy, Science and Technology, 540142 Targu Mures, Romania; 4 The School for Doctoral Studies in Biomedical Sciences, “Dunărea de Jos” University of Galați, 800008 Galati, Romania; g.alinaaa96@yahoo.com; 5 Department of Surgery, Clinical County Emergency Hospital, 810325 Brăila, Romania

**Keywords:** STUMP, leiomyoma, leiomyosarcoma, uterine smooth muscle tumors

## Abstract

Uterine smooth muscle tumors (USMTs) represent a diagnostically and clinically challenging subset of uterine mesenchymal neoplasms. Up to 5% of these tumors exhibit ambiguous histological features that preclude definitive classification as either benign leiomyomas or malignant leiomyosarcomas. This review provides a comprehensive synthesis of the evolving diagnostic criteria, histopathological variants, and recent advancements in immunohistochemical and molecular profiling of smooth muscle tumors with uncertain malignant potential (STUMPs). The review traces the historical development of diagnostic criteria, from the original mitotic thresholds to the “Stanford criteria,” which incorporate mitotic index, cytological atypia, and tumor cell necrosis. Contemporary WHO guidelines largely uphold these principles, with nuanced refinements for spindle, myxoid, and epithelioid subtypes. However, recent studies suggest additional morphologic indicators, such as atypical mitoses, infiltrative margins, and vascular invasion, may provide prognostic insight. Notably, necrosis remains the most reliable histologic predictor of recurrence, while mitotic activity and atypia, though important, are less specific. In conclusion, STUMPs represent a heterogeneous group with unpredictable behavior that requires long-term clinical follow-up. While existing histological and molecular tools aid classification, definitive prognostic markers remain elusive. Further studies integrating histopathology, immunohistochemistry, and molecular biology are essential to refine diagnosis and improve therapeutic decision-making in this diagnostically ambiguous group of uterine tumors.

## 1. Introduction

Uterine smooth muscle tumors (USMTs) represent the commonest mesenchymal neoplasms of the female genital tract, with a high incidence, estimated between 45% and 70% [1]. Among these, approximately 1–2% are leiomyosarcomas (LMS) and 45–65% are leiomyomas (LM) [1,2]. However, a small, yet clinically significant subset, accounting for 2–5% of cases, exhibits ambiguous morphologic features that preclude definitive classification as either benign or malignant [2,3,4,5]. According to the latest diagnostic criteria, these borderline lesions are considered smooth muscle tumors of uncertain malignant potential (STUMP) [3,4,5]. The term was introduced for the first time in 1973 to describe a clinically aggressive neoplasm in which, due to limited diagnostic capabilities at the time, a definitive histological diagnosis of LMS could not be established [6]. Despite their rarity, STUMPs pose significant diagnostic and clinical challenges, primarily due to their unpredictable biological behavior and limited histopathological criteria for risk stratification [2,3,7]. In recent years, immunohistochemical markers and molecular testing have been explored as adjuvants to conventional histology, yet no definitive algorithms have been validated [8].

This review aims to provide an updated synthesis of the literature regarding STUMP and its mimickers, with a focus on diagnostic criteria, histopathological subtypes, and ancillary immunohistochemical and molecular findings. By integrating current data, we emphasize the importance of long-term follow-up and the need for further studies to improve prognostic assessment and therapeutic decision-making.

## 2. Uterine Smooth Muscle Tumors Diagnostic Criteria in the Last 50 Years

The original criteria for a diagnosis of STUMP were (1) at least 15 mitoses/10 high-power fields (HPF), (2) 6–9 mitoses/10 HPF and minimal atypia in a tumor with increased cellularity, or (3) 3–5 mitoses/10 HPF in a tumor with cytological atypia [9]. Twenty-one years later, based on the observation that, using the original criteria, a significant percentage of USMTs were STUMPs, a pivotal study by Bell et al. addressed this problem and changed the way these tumors should be approached. In addition to the original features, tumor cell coagulative necrosis was taken into account as a criterion [10]. Since 1994, with the publication of Bell et al.’s research, the parameters identified as the most important criteria for prognostic assessment, known as “Stanford parameters”, are still used. These are (1) mitotic index, (2) significant atypia, and (3) coagulative tumor cell necrosis. When all these features are present or absent, a diagnosis of LMS or LM is straightforward. Moreover, even two out of the three aforementioned criteria are enough for a definite diagnosis of malignancy [11].

Based on the updated Bell’s parameters, these tumors with undetermined biological potential were grouped in four categories: (1) “atypical leiomyomas with limited experience” (AL-LE)—smooth muscle tumors without necrosis, with focal or multifocal moderate/severe atypia, with no more than 10 mitoses/10 HPF, (2) “smooth muscle tumor with low malignant potential” (SMT-LMP)—smooth muscle tumors with necrosis, with minimal or no atypia and no more than 10 mitoses/10 HPF, (3) “atypical leiomyoma with low risk of recurrence (AL-LRR)—smooth muscle tumors without necrosis, with diffuse, moderate/severe atypia and no more than 10 mitoses/10 HPF and (4) “mitotically active leiomyoma with limited experience (MAL-LE)—smooth muscle tumors without necrosis or atypia, but with at least 20 mitoses/10 HPF [10].

**Figure 1:**
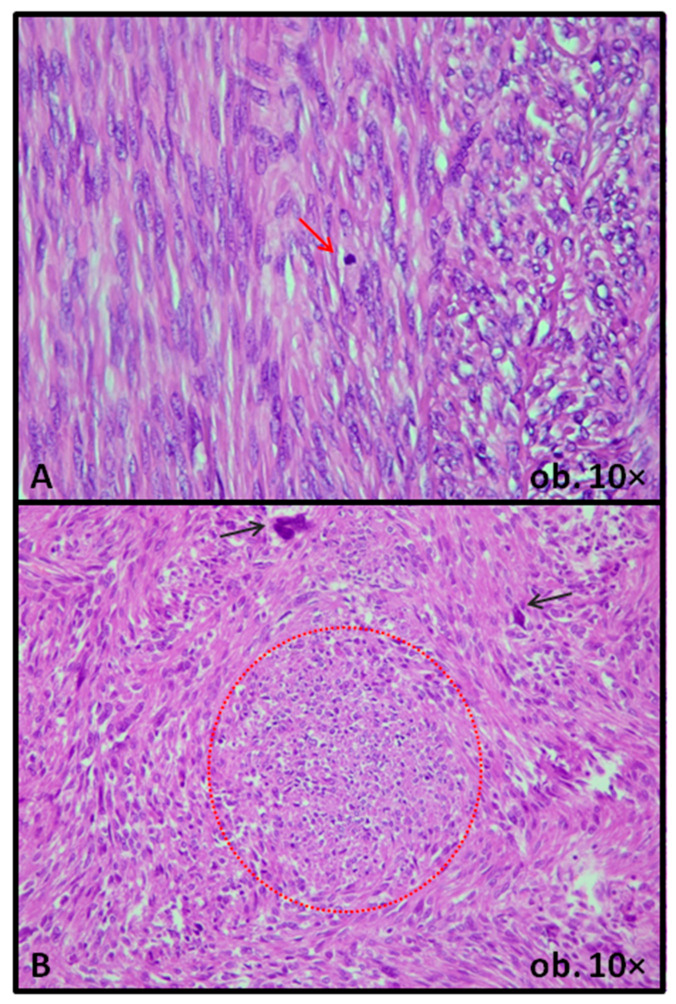
Smooth muscle cell tumor of uncertain malignant potential: scattered mitotically active nuclei (red arrow) (**A**), or atypical nuclei (black arrows), in the context of necrotic foci of undetermined origin (red circle) (**B**).

The current edition of the World Health Organization (WHO) manual of classification of Female Genital Tumors defines STUMP as “a tumor that shows morphological features that exceed criteria for LM or its subtypes, yet are insufficient for a diagnosis of LMS, and behaves in a malignant fashion in only a minority of cases” [11]. It recognizes three histological subtypes, specifically spindle, myxoid, and epithelioid STUMP. Regarding the diagnostic criteria, those proposed by Bell et al. more than 30 years ago are stated to be almost identical. Even though Bell et al. did not use in their study the term “STUMP”, naming these tumors “problematic smooth muscle cell tumors”, the only difference in criteria list from the current edition of WHO manual is a lower threshold of mitotic count from “at least 20/10 HPF” to “at least 15/10 HPF”, in case of the previously MAL-LE tumor. According to the WHO manual, these diagnostic parameters should be used only for STUMP with spindle morphology [11]. For the other two subtypes, the diagnostic criteria, according to the WHO manual, are stricter and refer to “all tumors that exceed the criteria for LM but fall below the threshold of LMS” [11]. The histologic heterogeneity of STUMPs, with either increased mitotic activity or cytologic atypia with foci of an undetermined type of necrosis, is illustrated in Figure 1.

According to multiple studies, the highest recurrence rate is observed in cases of STUMP diagnosed based on the presence of necrosis, even though these tumors do not exhibit, by definition, mitoses or cellular atypia [12,13,14,15]. Nevertheless, in many cases, a straightforward diagnosis of tumor cell coagulative necrosis is difficult. The most important hint for a diagnosis of non-tumor, ischemic type necrosis is the presence of granulation tissue, accompanied or not by fibrosis, at the junction between viable and necrotic tissue. In infarction-type necrosis, the necrotized cells exhibit a ghost-like appearance, including those adjacent to blood vessels. On the other hand, in tumor cell coagulative necrosis, granulation tissue and fibrosis are usually lacking, and in some areas, tumor cell viability is kept around blood vessels [10,11,16]. Because of this ambiguity, the most recent guidelines suggest a diagnosis of STUMP in cases with necrosis of undetermined origin [5,10,11,17] (Figure 1).

However, a high mitotic count or the presence of cytological atypia, independently, are not considered enough for a diagnosis of STUMP: USMT with only high mitotic count is, according to the current classification, diagnosed as mitotically active LM, while USMT with only cytological atypia should be diagnosed as LM with bizarre nuclei [11]. The terms “atypical leiomyoma” and “atypical smooth muscle neoplasm” are discouraged by the latest WHO edition [11].

A more recent study, published by Gupta et al., emphasized the cardinal role of the study by Bell et al., but, at the same time, reasonably raised the question of the necessity of expanding the histologic criteria for STUMP [14]. For example, the presence of nuclear atypia, independent of the presence of atypical mitoses, should be considered a putative marker for adverse prognosis. In their study, Gupta et al. demonstrated that out of eight patients with local recurrence or distant metastases, seven had tumors with either multifocal or diffuse cytological atypia. They considered the presence of cytological atypia a marker of adverse outcome, noting that its presence reflects genetic and epigenetic changes that develop during tumor progression. On the other hand, as opposed to necrosis, not all tumors with cellular atypia have a dismal prognosis. Hence, if necrosis is a feature both sensitive and specific for STUMP (if not LMS), cellular atypia should be regarded as a feature with good sensitivity, even though it lacks specificity [14] (Table 1).

Regarding the proliferation index, the same study agrees with Bell’s conclusions. Moreover, in their cohort, they even demonstrated that the mitotic count mean was slightly lower in cases with an adverse outcome, compared to those with a favorable outcome. Hence, even though the mitotic count is an important prognostic predictor, it does not have a significant impact if it is not associated with another feature of poor prognosis, such as cytological atypia [14].

Other morphologic features that were not addressed in Bell’s study and that might help distinguish benign from malignant biological behavior in STUMPs include atypical mitoses, poorly circumscribed/infiltrative margins, vascular space involvement, and focal epithelioid morphology, insufficient for a diagnosis of epithelioid STUMP. They were not included as diagnostic tools for STUMP because most of them were considered features of LMS at the time. More recent studies confirmed the possibility of infiltrative margins, epithelioid morphology, atypical mitoses, and even vascular space involvement in smooth muscle tumors with a benign clinical outcome [14,15,16,17].

In their study, Guntupalli et al. included 41 patients diagnosed with STUMP and classified tumors into five categories based on necrosis, mitotic count, degree of atypia, cellularity, presence of irregular tumor margins, and peripheral vascular invasion [17].

D’Angelo et al. propose, similarly to Bell’s study, four sets of criteria, using the same parameters, necrosis, atypia and mitotic count: necrosis difficult to classify, focal or diffuse, marked atypia in a tumor with 8–9 mitoses/10 HPF, undetermined necrosis in a tumor with at least 10 mitoses/10 HPF and tumor necrosis in a typical LM [18].

Nonetheless, in tumors in which classic, accepted criteria for STUMP are not met, and still the pathologist is not comfortable with a diagnosis of LM, supplementary morphologic features, such as a small proportion of tumor cells with epithelioid morphology, or an infiltrative pattern of growth, could guide the diagnosis of STUMP [8,17,19].

Historically, this entity has been characterized by increased heterogeneity, whether we are talking about incidence, prevalence, or the percentage of cases with adverse outcomes [3,7,14,18]. This is due primarily to changes in diagnostic criteria over time. For example, the formerly named AL-LRR (atypical leiomyoma with low recurrence risk) tumor, included in Bell’s study as “problematic smooth muscle tumors”, latter studies confirmed a much lower risk for recurrence, compared to other categories, reason why the accepted nomenclature today groups this tumor in the category of LM, specifically LM with bizarre nuclei [11].

## 3. Epithelioid and Myxoid Morphology in Uterine Smooth Muscle Tumors

While most uterine smooth muscle tumors have a classic spindle cell morphology, up to 5% of them exhibit epithelioid or myxoid differentiation [11]. Nevertheless, in the category of STUMP, these two particular morphologies are even rarer, with very few cases reported in the literature. Due to its paucity, there is a lack of specific, universally accepted criteria for diagnosing STUMP with epithelioid or myxoid morphology. According to the latest edition of the WHO manual, the essential diagnostic criteria for uterine LMS with epithelioid or myxoid morphology are stricter: only one feature of atypia is sufficient for diagnosis, unlike classic LMS, which requires at least two criteria [11] (Table 2).

From our perspective, given the low number of criteria needed for a diagnosis of malignancy in myxoid USMT, myxoid STUMP is virtually inexistent [11,20,21]. There is, from our point of view, no gap between the criteria of benign and malignancy in case of USMT with myxoid features, at least from the morphologic point of view. With regard to clinical behavior and outcome, the discussion is more complex, because not all myxoid USMTs, even if they are diagnosed as LMS, will exhibit a malignant clinical behavior and associate a poor outcome.

**Figure 2:**
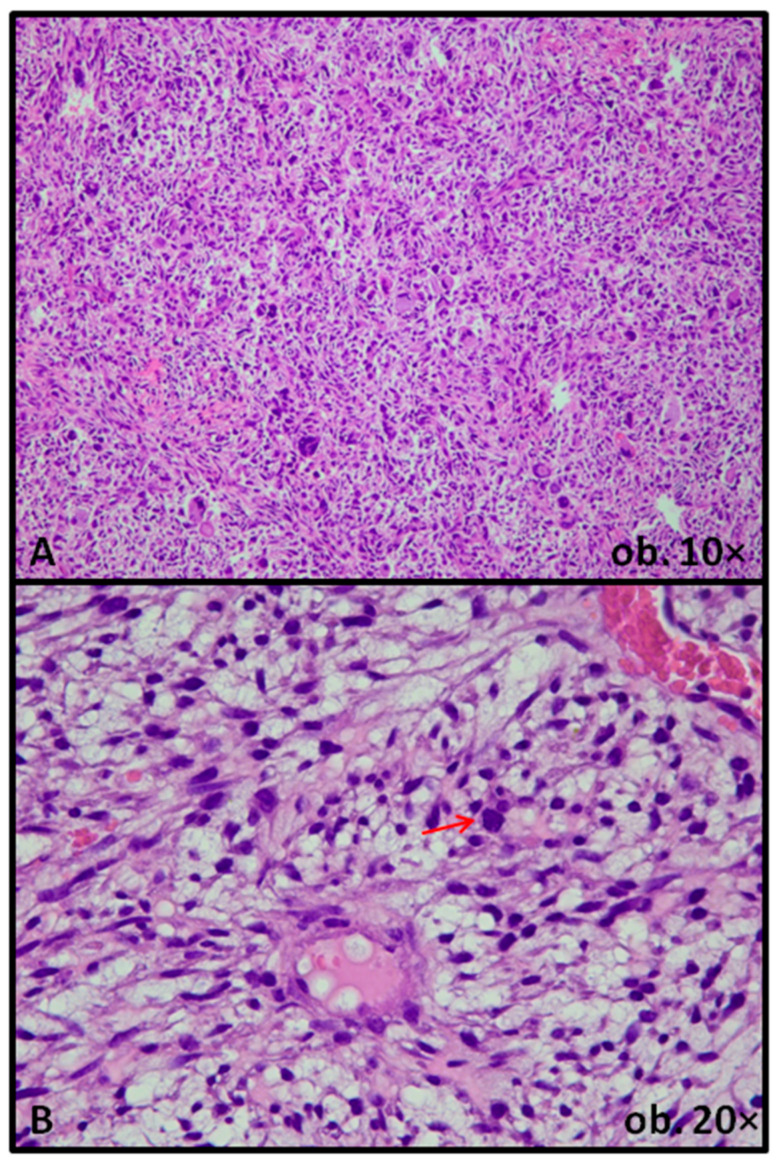
Leiomyosarcoma: conventional variant, with atypical pleomorphic cells and mitotically active nuclei (**A**), and myxoid variant, exhibiting more subtle features of malignancy, with rare mitoses (red arrow) (**B**).

Uterine LMSs, as illustrated in Figure 2, are defined as malignant USMTs showing at least moderate to severe cytologic atypia, elevated mitotic activity (traditionally ≥10 mitoses/10 high power fields), and coagulative tumor cell necrosis. Myxoid LMSs represent a variant in which atypical smooth muscle cells are embedded in abundant myxoid stroma and may display deceptively low mitotic counts; therefore, the diagnosis relies on the combination of nuclear atypia, infiltrative growth, and coagulative tumor cell necrosis rather than mitotic index alone [20,21].

A morphology similar to that of myxoid, which can sometimes be confused by inexperienced pathologists with true myxoid differentiation in USMTs, is the so-called hydropic change [11]. While the myxoid aspect appears due to a secretion and accumulation of glycosaminoglycans rich in hyaluronic acid, the “pseudomyxoid” or hydropic change is simply a reactive change due to accumulation of transudate [21]. The latter has no clinical significance, reason why it is of paramount importance a definite and correct differentiation between these two types of morphologic changes, especially in tumors in which only one criteria suggestive of poor outcome, such as irregular tumor margins, cellular atypia, or mitotic activity, is present: a true myxoid differentiation in such tumor will lead to a diagnosis of LMS, while hydropic change, in the same context, will sustain a diagnosis of LM, not even of STUMP [20,22]. A particular type of LM with irregular, pseudoinfiltrative margins and commonly exhibiting hydropic changes is the so-called cotyledonoid or dissecting LM. In cases in which a diagnosis of myxoid or hydropic change cannot be made on H&E stain, additional stains, such as Alcian blue, can be used [22,23,24].

## 4. Benign Uterine Smooth Muscle of the Uterus

**Figure 3:**
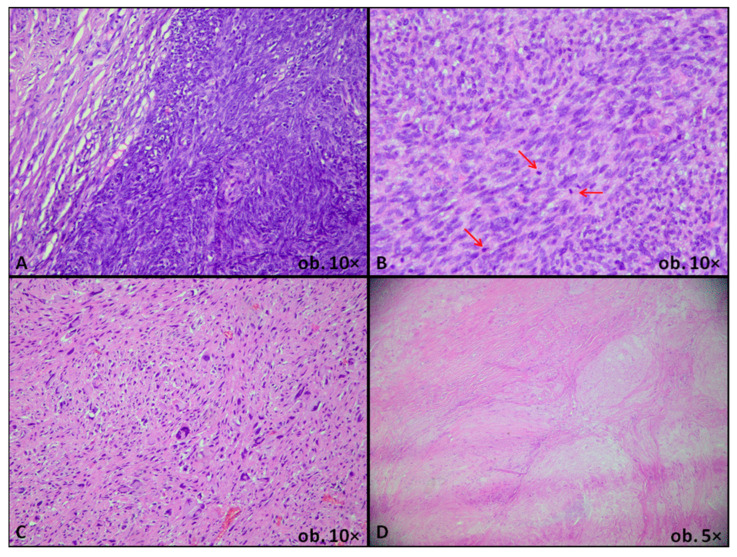
Histologic variants of leiomyoma: cellular leiomyoma—well circumscribed smooth muscle cell proliferation with dense cellularity, without atypia (**A**); mitotically active leiomyoma—smooth muscle cell proliferation with scattered typical mitoses (red arrows), without necrosis or atypical cells (**B**); leiomyoma with bizarre nuclei—smooth muscle cell proliferation with multiple bizarre nuclei, without necrosis or atypical mitoses (**C**); cotyledonoid-dissecting leiomyoma—multinodular, dissecting smooth muscle proliferation with hydropic stroma and bland cytologic features (**D**).

Benign USMTs (LMs) are the most frequent mesenchymal neoplasms of the uterus. According to the current WHO classification, this group includes conventional LM and several histologic variants, each defined by distinct morphologic features and clinical behavior [11]. Conventional LM is a well-circumscribed, fascicular proliferation of bland smooth muscle cells with cigar-shaped nuclei, inconspicuous nucleoli, a low mitotic index, and absence of coagulative tumor necrosis. Several histologic variants have been described. These include cellular LM, characterized by marked hypercellularity with bland cytology; mitotically active LM, which shows typical morphology and, as its name implies, an increased mitotic index, and LM with bizarre nuclei, defined by focal areas of markedly pleomorphic or multinucleated cells within an otherwise typical LM. Additional variants include cotyledonoid-dissecting LM, characterized by nodular, exophytic or intramural dissecting growth, often associated with hydropic change yet retaining benign cytological features; hydropic LM, with conspicuous edematous stroma compartmentalizing smooth muscle bundles, and apoplectic LM, showing foci of infarction, with hypercellular bands around them; lipoleiomyoma contains a variable mature adipose component, whereas epithelioid LM is composed predominantly of round or polygonal cells with eosinophilic or clear cytoplasm. Fumarate hydratase-deficient LM is characterized by distinctive nuclear atypia and prominent nucleoli and is associated with hereditary leiomyomatosis and renal cell carcinoma in a subset of patients. This distinct WHO-recognized subtype is typically driven by biallelic *FH* inactivation. Morphologically, it shows increased cellularity with atypical or bizarre nuclei and prominent eosinophilic nucleoli with perinuclear clearing. Immunohistochemistry usually demonstrates loss of FH expression. Because a subset of patients has germline *FH* variants, recognition should prompt consideration of genetic counseling and renal surveillance in appropriate clinical contexts [11]. Finally, diffuse uterine leiomyomatosis is defined by multiple coalescent nodules that diffusely expand the myometrium [11,25]. As illustrated in Figure 3, recognition of these variants is essential, as their accentuated cellularity, nuclear atypia, unusual growth patterns, or alarming gross appearance may closely mimic STUMP or LMS. Nevertheless, the combination of low mitotic activity, absence of tumor cell necrosis, and typically pushing rather than infiltrative margins, together with correlation with clinical and radiologic findings, supports a diagnosis within the LM spectrum according to WHO-based criteria and contemporary expert reviews [7,11].

## 5. Benign Uterine Smooth Muscle Tumors with “Pseudomalignant” Features

Despite their clearly benign morphology, a subset of USMTs can, in rare instances, exhibit extrauterine involvement. These entities are benign metastasizing leiomyoma (BML) and disseminated peritoneal leiomyomatosis (DPL) [11]. BML is defined as one or more nodules of histologically benign smooth muscle cells occurring at sites distant from the uterus, most typically affecting women of reproductive or perimenopausal age with a previous history of uterine LM treated by myomectomy or hysterectomy. Histologically, the metastatic nodules closely resemble conventional LM, showing bland spindle cells, low mitotic activity, absence of coagulative necrosis, and a typical smooth muscle immunophenotype with diffuse SMA expression. Cytogenetic and molecular studies have demonstrated identical alterations in uterine and metastatic nodules, supporting a clonal relationship and a true metastatic process rather than a multifocal de novo proliferation [26]. Rare cases affecting the brain or the abdominal organs have been described. Clinically, BML usually follows an indolent, hormone-sensitive course; therefore, management is based on surgery and hormone treatment, with long-term imaging follow-up to exclude progression or transformation [26,27,28,29,30,31]. In addition, intravenous leiomyomatosis (IVL) could be included in this category [26,27,28,29]. Regarding the underlying mechanism of BML development, published data remain partially contradictory. Some studies suggest a common origin for BML and conventional uterine LM, supported by shared molecular pathways and genetic alterations [32,33,34]. Other authors have proposed a distinct genetic background, as BML may exhibit chromosomal loss 3q, 11q, 19q, and 22q, alterations typically absent in conventional LM [35].

DPL is a condition in which, on the surface of the peritoneum and omentum, multiple nodules are scattered, having variable diameters, but most of them are less than 1 cm [36,37,38]. In the cut section, these tumorlets exhibit a classic, whorled/fascicular aspect, similar to conventional myometrial LM. Similarly, the microscopic aspect is overtly benign, with no atypia, mitotic activity, or necrosis. This condition seems to be induced by increased hormone levels, being described in pregnant women or in perimenopausal women with estrogen-substitutive therapy [39,40,41]. Genetic studies identified a specific pattern of X chromosome inactivation, present in all cases, suggesting a clonal origin [41,42,43]. Other hypotheses support a metaplastic origin, with mesenchymal or smooth-muscle metaplasia of the submesothelial cells, in response to hormonal stimuli [44,45]. IVL is defined by the presence of an otherwise typical LM in vascular spaces within the uterine wall, in some cases with distant extension in the cava vein or even within the right atrium [43,46,47,48]. Even though the lesion is not categorized as malignant, it can be associated with various systemic symptoms, depending on its extent: Cava vein or atrial involvement can produce dyspnea and syncopal episodes. This entity seems to share some of the genetic abnormalities with typical LM, yet *MED12* mutations, a commonly seen alteration in classic LM, have not been documented in IVL [49].

Recent advances in molecular pathology have significantly contributed to the understanding and classification of USMTs, offering valuable insights into their biological behavior and helping refine diagnostic accuracy. Traditional histopathologic evaluation remains the cornerstone for diagnosing USMTs; however, it is increasingly supplemented by molecular and genetic findings that can help differentiate between benign, uncertain, and malignant categories. Recent studies have identified key molecular alterations that may help stratify LM, STUMP, and LMS. For instance, *MED12* mutations are frequently found in conventional LM but are less commonly seen in STUMP and LMS. In contrast, *TP53* mutations, *RB1* deletions, and chromosomal instability are more characteristic of LMS, supporting their aggressive clinical course [50,51].

From a comparative perspective, USMTs show a partially distinct molecular spectrum when compared with LMs and LMS arising in soft tissue. Most uterine LMs fall into a limited number of mutually exclusive molecular subgroups defined by *MED12 exon 2* mutation, *HMGA2/HMGA1* rearrangements, biallelic inactivation of fumarate hydratase (*FH*-deficient leiomyoma), or, less commonly, *COL4A5-COL4A6* deletions, whereas these alterations are much less frequent in extrauterine LMs. In contrast, uterine and soft tissue LMSs share similar complex karyotypes with widespread copy-number alterations affecting cell-cycle and DNA damage response pathways, but recent methylation and transcriptomic studies suggest that uterine LMS form a molecularly distinct subgroup within the broader family of LMSs. These site-related differences underline the importance of considering anatomic location when interpreting molecular data and extrapolating biomarkers validated in soft-tissue sarcomas to USMTs [52,53].

In addition to clarifying the spectrum of USMTs, recent research has drawn attention to the diagnostic challenges in distinguishing these tumors from other uterine mesenchymal tumors, particularly inflammatory myofibroblastic tumors (IMT), often present as spindle cell lesions with fascicular or myxoid areas and variable mitotic activity, sometimes resembling STUMPs or low-grade LMS. However, IMTs typically show a prominent inflammatory infiltrate and are characterized by *ALK*, *ROS1*, or other kinase rearrangements, with corresponding *ALK/ROS1* expression on immunohistochemistry or FISH, findings that are not seen in conventional USMTs. Perivascular epithelioid cell tumors (PEComas) can also mimic LMS, particularly the epithelioid variants, because they may express SMA or desmin. Nevertheless, they usually display a perivascular growth pattern and co-express melanocytic markers such as HMB45, Melan-A, and cathepsin K, a unique immunophenotype absent in LM, STUMP, or LMS [54,55].

Solitary fibrous tumor (SFT) of the uterus is rare but enters the differential diagnosis as a spindle-cell lesion with a patternless architecture and branching, staghorn vessels. SFTs are typically CD34-positive and show nuclear STAT6 expression, while smooth muscle markers are negative or only focally expressed [56].

Gastrointestinal stromal tumors (GISTs) involving the pelvis can mimic LMS but usually arise from the bowel and show diffuse KIT and DOG1 expression, with only limited desmin and h-caldesmon staining [57].

Because *FH*-deficient LMs can display atypical features, they may be overdiagnosed as STUMP, particularly when borderline cytologic atypia is present in the absence of unequivocal malignant criteria. In STUMP-like USMT with morphologic clues suggestive of *FH* deficiency, adding FH immunohistochemistry can help reclassify a subset of cases within the *FH*-deficient LM spectrum and guide appropriate clinical triage for possible hereditary leiomyomatosis and renal cell carcinoma [58].

Importantly, *FH* deficiency is not restricted to benign USMTs. A large cohort study (*n* = 348) demonstrated immunohistochemical evidence of FH deficiency in ~2% of uterine LMS, including complete FH loss with diffuse 2-succinocysteine (2SC) positivity and occasional subclonal patterns. Macronucleoli with perinucleolar clearing were significantly enriched among *FH*-deficient uterine LMSs, underscoring that *FH* loss and diffuse 2SC positivity do not exclude malignancy and must be interpreted strictly in conjunction with established morphologic criteria, such as tumor cell necrosis, mitotic activity, and infiltrative growth [59].

Finally, endometrial stromal sarcomas (ESS) and undifferentiated uterine sarcoma may present as high-grade spindle or pleomorphic neoplasms infiltrating the myometrium and can be misdiagnosed as LMS. Low-grade ESS is composed of small stromal cells with characteristic arteriolar-type vasculature, strong CD10 and ER/PR expression, whereas high-grade ESS and undifferentiated uterine sarcoma often lack smooth muscle markers but express cyclin D1 or show diffuse aberrant p53 and p16 expression [60]. These tumors may closely resemble USMTs both morphologically and immunohistochemically, leading to potential diagnostic pitfalls. In all these settings, correlation of morphology with a targeted immunohistochemical and molecular work-up is essential to correctly classify the tumor and separate USMTs from their mimics [61].

## 6. Ancillary Testing in Uterine Smooth Muscle Tumors

With the development of immunohistochemistry techniques, a great step forward was made in the field of pathology—a more precise diagnosis can be established, and also, the prognostic and predictive impact was enhanced. In USMTs, the cell of origin/the line of differentiation can be easily diagnosed in most cases on H&E, with the need for confirmatory IHC stains being limited. Nevertheless, in some tumors with particular morphology—epithelioid or myxoid, specifically in malignant types, the smooth muscle differentiation can be confirmed by h-caldesmon, SMA, muscle-specific actin, or desmin staining. Novel markers, such as histone deacetylase 8 (HDAC8) and myocardin, have proven to be more specific for smooth muscle differentiation in epithelioid tumors [61,62].

Thus, the best use of IHC in these tumors remains to predict prognosis. Nevertheless, the potential of IHC biomarkers in this field remains controversial. Among the most studied are p16, p53, ki67, Twist, bcl2, p21, CD117, and hormone receptors for estrogen, progesterone, and androgen. From those, the most promising results were observed with p16, p53, and ki67. In LMS, p16 overexpression is present in more than 70% of cases [13,63,64]. One study even suggested that p16 overexpression can serve as an indicator of malignancy in STUMP associated with necrosis [17]. On the other hand, p16 positivity can also be observed in particular subtypes of LM, such as LM with bizarre nuclei/symplastic LM or apoplectic LM, in which hypercellular areas can display a positive, overexpressed pattern, which limits its usefulness in some cases [62]. Regarding p53 expression, most studies reached similar conclusions: Overexpression in more than 50% of tumor cells is an indicator of poor outcome, whereas a lack of p53 staining is not always suggestive of good prognosis [64,65,66,67,68,69,70]. Ki67, used as a proliferative index, is overexpressed in classic types of USMTs and in LMS, with a cut-off around 30%. Nevertheless, a similar percentage can be present in STUMP with poor outcome or in particular types of LM—mitotically active type and, similar to p16, in hypercellular areas of apoplectic-type smooth muscle tumors [64,71,72,73,74].

Although these markers are helpful, there is currently no single immunohistochemical or molecular biomarker that can unequivocally distinguish LM, STUMP, and LMS. Conventional LMs usually show diffuse strong desmin and h-caldesmon positivity, strong ER/PR expression, a wild-type p53 staining pattern, and a very low Ki-67 index. STUMPs generally retain a LM-like immunoprofile but may display intermediate Ki-67 labeling and focal or patchy p16 expression, without the diffuse, aberrant p53 pattern typical of LMS. In contrast, LMSs frequently exhibit high proliferative activity (>20–30% Ki-67), diffuse strong p16 expression, mutant-type p52 staining, and often reduced ER/PR expression. Therefore, immunohistochemical panels are best used as supportive tools and must always be interpreted in conjunction with the key morphological criteria when classifying USMTs [8].

## 7. MicroRNA (miRNA) Network in Uterine Smooth Muscle Tumors

USMTs, especially the malignant counterpart (LMSs), are known to harbor an extremely unstable genetic background, with complex chromosomal changes [72,73,74,75]. Among those, the vast majority are somatic mutations; only one, the *FH* gene, is specifically linked to smooth muscle tumorigenesis, encoding a protein called fumarate hydratase [76,77,78,79,80]. In addition to smooth muscle tumors, *FH* gene mutation harbors also a risk for renal tumors, especially renal cell carcinoma [78].

While the genetic alterations involved in the occurrence of USMTs are described in many studies, the epigenetic events that could play a role in tumor initiation are poorly understood to date.

In the field of epigenomics, one of the most important regulatory mechanisms, with an increasingly recognized role, is the microRNA machinery. miRNAs are small non-coding RNA molecules, typically 18 to 25 nucleotides in length, that regulate gene expression at the post-transcriptional level [81]. They function by binding to complementary sequences in the untranslated region of messenger RNA, leading to its degradation or translational repression. miRNAs play essential roles in numerous biological processes, such as cell development, cell differentiation, apoptosis, and disease mechanisms, including tumors [81,82].

To confirm the hypothesis according to which the molecular mechanisms involved in the occurrence of each type of USMT—LM, STUMP, and LMS—do not overlap, we searched synthetically post-transcriptional signatures at the miRNA level for each entity and compared them. Our approach was to construct a miRNA network for each tumor type. For this, we used miRNet online, a free-access platform [83].

**Figure 4:**
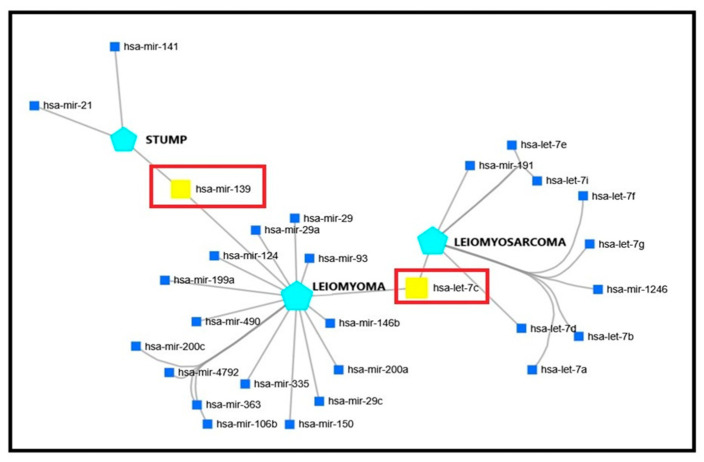
miRNA network in uterine smooth muscle tumors, emphasizing the different patterns of miRNA expression in LM, STUMP, and LMS: only one miRNA is common for LM and STUMP molecular network (has-mir-139), and one for LM and LMS (hsa-let-7c), without any common miRNA for STUMP and LMS (generated by using miRNet online, free-access platform).

There were 28 miRNAs involved in LM/STUMP/LMS network, most of them regulating the LM molecular machinery: hsa-miR-29, hsa-miR-29a, hsa-miR-124, hsa-miR-199a, hsa-miR-490, hsa-miR-200c, hsa-miR-4972, hsa-miR-363, hsa-miR-106b, hsa-miR-335, hsa-miR-150, hsa-miR-29c, hsa-miR-200a, hsa-miR-146b, hsa-miR-93, hsa-miR-139 and hsa-let-7c. LMS pathway is regulated by 10 miRNAs, 8 of them from the lethal-7 (let-7) miRNA family: hsa-let-7a, hsa-let-7b, hsa-let-7c, hsa-let-7d, hsa-let-7e, hsa-let-7f, hsa-let-7g, and hsa-let-7i, the other 2 being hsa-miR-191 and hsa-miR-1246. Ultimately, STUMP-network was least represented, with only 3 miRNAs as modulators: hsa-miR-21, hsa-miR-141, and hsa-miR-139 (Figure 4).

## 8. Clinical Management

Following a diagnosis of STUMP, clinical management becomes particularly important due to the rare and unpredictable nature of this tumor. STUMP represents a histological gray zone between LM and LMS. Although these tumors often behave in a benign manner, they carry a small but real risk of recurrence or progression, including rare cases of metastasis or transformation into LMS [84,85,86,87,88,89,90]. Given this uncertainty, there is no universally accepted protocol for post-treatment follow-up, and management strategies are largely informed by small case series, retrospective studies, and expert opinion.

Current clinical practice typically recommends conservative but vigilant surveillance following surgical excision, particularly in women of reproductive age who wish to preserve fertility. Myomectomy is often the preferred surgical approach for such patients, while hysterectomy may be considered in post-reproductive women or in cases with concerning pathological features. Regardless of the surgical approach, long-term follow-up is essential due to the possibility of late recurrence, which has been reported several years after initial resection [91].

Postoperative follow-up generally includes periodic clinical evaluation and imaging. Pelvic examination combined with imaging techniques—particularly transvaginal ultrasound and pelvic MRI—forms the cornerstone of surveillance. MRI is often favored in these cases due to its superior soft-tissue resolution, which allows for better differentiation between scar tissue, recurrent tumors, and other pelvic masses. Follow-up intervals vary among clinicians but are typically scheduled every 6 to 12 months for the first 3 to 5 years, with some experts recommending continued annual surveillance thereafter [92].

The surveillance should be intensified in cases with high-risk histological features, such as high mitotic activity, tumor necrosis, cytologic atypia, or infiltrative margins. In addition to imaging, some clinicians may consider using laboratory tests or biomarkers, although their utility in STUMP is currently limited and not well established. In rare cases where recurrence is suspected or malignant transformation is possible, more advanced imaging modalities like PET-CT or CT scans may be employed to assess for distant disease. However, routine use of these tools is not typically recommended unless clinically indicated. The lack of standardized guidelines means that management decisions must often be individualized. Factors such as patient age, desire for future fertility, tumor extent, completeness of surgical excision, and pathologic features all influence the follow-up plan. Patients should be fully counseled about the uncertain nature of STUMP and the importance of long-term monitoring, even in the absence of symptoms [91,92].

## 9. Conclusions

Uterine smooth muscle tumors of uncertain malignant potential (STUMP) remain a major diagnostic challenge, lying at the intersection between benign and malignant entities. Their variable histopathological features, the absence of standardized IHC markers or molecular diagnostic tools, and their unpredictable biological behavior significantly limit the ability to accurately assess prognosis and guide clinical management. Although numerous IHC and molecular assays have been proposed to aid in risk assessment, none have yet achieved universal acceptance or validation for routine diagnostic use. In this context, a multidisciplinary approach and long-term clinical surveillance are essential.

## Figures and Tables

**Table 1:** Comparative microscopic features of smooth muscle tumor of uncertain malignant potential (STUMP) and leiomyosarcoma (LMS).

Histopathologic Feature	STUMP	LMS
Tumor necrosis	Absent or present but associated with <4 mitoses/10 HPF and mild/moderate atypia	Present
Mitotic index (mitoses/10 HPF)	<10 (5–9, but without other malignant features)	≥10
Nuclear atypia	Mild/moderate (or severe, but without other malignant features)	Severe
Infiltrative borders	Absent (if present, not associated with other malignant features)	Present, clearly infiltrative
Ki67 index	Variable, if increased, still <10%	Frequently >30%
p53	Usually wildtype (non-mutant)	Usually mutant phenotype
p16	Normal expression	Usually overexpressed
Biological behavior	Unpredictable	Aggressive

**Table 2:** WHO criteria for uterine leiomyosarcoma [11].

	Cytological Atypia	Tumor Cell Necrosis	Mitotic Index	Infiltrative Margins/Irregular Borders
Spindle cell uterine leiomyosarcoma (2 or more of the following features)	2+/3+ nuclear atypia	Present	≥10 mitoses/10 HPF	/
Epithelioid uterine leiomyosarcoma (1 or more of the following features)	2+/3+ nuclear atypia	Present	≥4 mitoses/10 HPF	/
Myxoid uterine leiomyosarcoma (1 or more of the following features)	2+/3+ nuclear atypia	Present	≥1 mitosis/10 HPF	Present

## Data Availability

No new data were created or analyzed in this study.
